# Magnitude, response, and psychological determinants of placebo effects in chronic low-back pain: a randomised, double-blinded, controlled trial

**DOI:** 10.1097/PR9.0000000000000744

**Published:** 2019-06-07

**Authors:** Damien Finniss, Michael Nicholas, Charles Brooker, Michael Cousins, Fabrizio Benedetti

**Affiliations:** a Pain Management Research Institute, Royal North Shore Hospital, University of Sydney, Sydney, Australia; b Department of Anaesthesia, Royal North Shore Hospital, St Leonards, Australia; c School of Allied Health Sciences, Griffith University, Brisbane, Australia; d Medicine and Physiology of Hypoxia, Plateau Rosà, Italy/Switzerland; e Neuroscience Department, University of Turin Medical School, Turin, Italy

**Keywords:** Placebo, Placebo effect, Expectancy

## Abstract

**Introduction:**

Denervation of the lumbar zygapophyseal joints by medial branch radiofrequency neurotomy has shown some benefit in treating chronic low-back pain. Before denervation, a diagnosis is made by one or more blinded injections on separate occasions to ascertain whether the relevant joints are contributing to the pain. Placebo injections have been advocated in a diagnostic regime that also includes local anaesthesia, with a decision to proceed to neurotomy based on response to local anaesthesia and not to placebo.

**Objectives:**

We investigated the magnitude of and response rate to placebo injections, and the roles of expectation, desire for pain relief, and anxiety as determinants of response to placebo.

**Methods:**

One hundred twenty patients were randomised to receive placebo and local anaesthetic injections on alternate occasions in a double-blind manner. A smaller control group with 2 local anaesthetic injections was also used. Responses to placebo were characterised, including magnitude and frequency.

**Results:**

This study demonstrated very large response to placebo injections, both response rate (78%) and magnitude (effect size d = 1.85). Expectation and anxiety were important modulators of response to placebo in this setting, with support given to expectation as a dynamic modulator of placebo responses. Large response to placebo (both in rate and magnitude) was observed when participants reported the belief that they were in the placebo arm.

**Conclusion:**

This study demonstrated large placebo responses in the context of injections for low-back pain and further characterised the importance of expectation and anxiety as important psychological mediators.

## 1. Introduction

Chronic low-back pain (CLBP) is a global health issue, with significant burden at both an individual and societal level.^11^ There are many methods for diagnosis and treatment, one of which is to isolate nociceptive contributors to CLBP using a biomedical framework.^10,29^ Diagnostic injection studies have demonstrated the lumbar zygapophyseal joint (Z-joint) as a source of pain in 10% to 40% of patients.^30,32^ Controlled studies of subsequent denervation of the zygapophyseal joints (Z-joints) by medial branch radiofrequency neurotomy have shown benefit in some patients,^6,19^ although there have been negative trials.^35^ There is variability in the diagnostic injection process, both with the agents used and the criteria for a positive response, but ultimately the goal is to establish successful reduction in pain with local anaesthesia.^29^ The variability represents attempts to reduce false-positives (primarily placebo responses), and different paradigms have been advocated using single or multiple injections on repeated clinic visits, and with either local anaesthesia or placebo,^5,33^ although the use of placebo as a diagnostic tool has been questioned on ethical grounds by some organisations.^34^ A response to placebo is deemed to indicate a negative diagnostic block (ie, a placebo response rather than a “genuine” response, and the patient is not offered the neurotomy treatment).^5^ The use of this diagnostic regime in routine practice, with support from several guidelines, provided an opportunity to assess response to placebo injections in a clinical population who were already receiving placebo injections as part of their care.

A large proportion of studies on placebo effects, particularly placebo analgesia, has been conducted in experimental pain.^7^ Much of the data about magnitude of placebo effects have either come from experimental populations or from the control arms in clinical trials.^1,13^ In the latter, meta-analyses demonstrate a modest magnitude (standardised mean difference d = 0.15–0.27),^28^ whereas if only studies dedicated at assessing placebo effects are considered, the magnitude is higher (d = 0.51–0.95).^12,36^ Given there are multiple placebo effects with different determinants,^9^ and that these effects are context specific, it is not surprising that variance exists with regard to magnitude.

A similar issue is seen with understanding response rates. Early studies were limited in methodology, and no consistent predictors of a placebo responder have been identified.^14^ The inability to find a consistent “responder” is explained at a biological level (where multiple effects occur across many conditions)^14^ and supported by observations in trials that do not demonstrate consistent patterns of response; that is, that a subject will respond on one occasion and not another.^14^ In fact in one trial in this specific population, placebo responders were removed after response to the diagnostic placebo injections (leaving only those who responded to local anaesthesia); however, in the second arm of the study, a small percentage of “placebo nonresponders” responded to placebo RF neurotomy.^18^ Taken together, this suggests that assessment of response to placebo, and magnitude is context specific and requires more investigation.

Possible psychological determinants of placebo responsiveness in pain have been studied as well, in particular, expectancy. Expectancy is a person's confidence in their likely experience of an outcome or expected effect,^28^ and in the setting of placebo effects, expectancy refers to an individual's confidence in their likely response to treatment. Expectancies are context specific and can affect both pain intensity and emotional processing.^9,27^ There is evidence from studies with experimental pain,^2^ selected clinical populations using an experimental pain model (eg, irritable bowel syndrome),^37,39^ and postoperative pain^3^ that expectancy accounts for a significant proportion of the variance in postplacebo administration pain scores. Although expectancy is an established mediator of placebo effects, it is not the only mediator.^28^ Desire for symptom reduction and expectancy interact and underlie human emotions such as anxiety,^23–25^ and this interaction has predicted the magnitude of placebo analgesic responses.^37–39^ Furthermore, support exists for a close relationship between expectancy, desire and emotion (eg, anxiety), and magnitude of placebo analgesia.^37–39^ Taken together, these factors may operate independently or in combination in mediating placebo analgesia. But these relationships have had limited exploration in clinical pain.^9^

This study was intended to investigate the magnitude and response rate to placebo injections and the roles of expectancy, desire for pain relief, and anxiety in the placebo response. This study is part of a larger project assessing the utility of placebo injections as a diagnostic and prognostic tool (Finniss et al. in preparation).

## 2. Methods

### 2.1. Design

This study was a prospective randomised controlled trial of the diagnostic injection process in participants referred to a pain management centre for assessment and management of chronic lumbar Z joint pain. Ethics approval was granted by the Northern Sydney Health District's Human Research Ethics Committee. Clinical trial registration: ACTRN1217000622303.

### 2.2. Participants

One hundred and twenty participants were recruited during a 4-year period. The participants had been referred to the tertiary pain centre by either general medical practitioner or specialist. There was no formal advertising of the trial, although specialists who regularly refer to the clinic were notified of the trial. Participants were included if they were 18 to 80 years, had no signs or symptoms of sinister pathology and a clinical picture of pain originating from the lumbar z joints warranting further diagnostic injections and possible ongoing neurotomy treatment.^8^ Participant baseline characteristics are presented in Table 1 and are compared with published normative data for CLBP presenting to a tertiary referral pain centre.^21^ Interventions were conducted in a single hospital clinic.

**Table 1:**
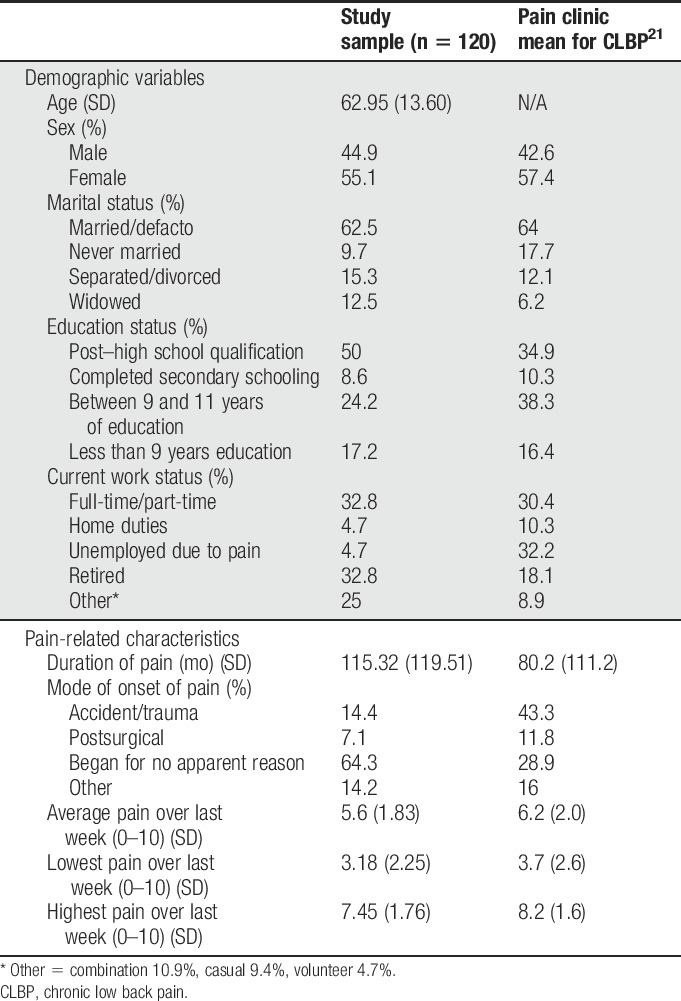
Baseline characteristics.

### 2.3. Interventions

**Figure 1.**
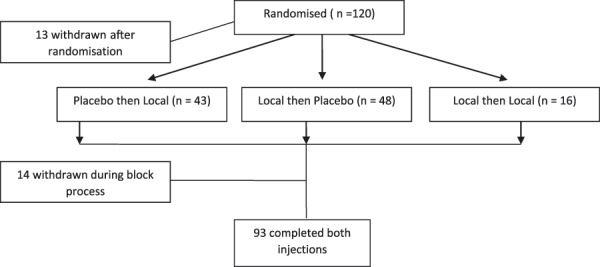
Participant flow.

After enrolment, participants were randomly assigned to 1 of 3 groups (Fig. 1). Group 1 received a placebo injection on the first injection occasion and a local anaesthetic (bupivacaine) on the second. Group 2 received the injections in the opposite order. Group 3 (the control group) received local anaesthetic on both visits. This group was intended to control for possible changes in expectancy after the first injection, specifically, that it was possible to have significant relief after the first injection and still have the possibility of receiving a local anaesthetic on the second. For example, after a successful first injection, the participant might expect that the second would be a placebo, and the blinded nature of the injection could be compromised. This group also controlled for biased reporting or guessing, as a participant could report benefit in the second block after poor response in the first in the hope of receiving a diagnosis and the neurotomy treatment.^5,31^ The possibility of receiving 2 identical local anaesthetic agents provided a simple way of controlling for these problems.

Participants attended the clinic on 2 separate occasions for diagnostic injections. These visits were a minimum of 1 week apart and were often several weeks apart. On the first occasion, the message given to each participant was that there was a possibility of receiving a local anaesthetic or a placebo injection, and that a response to either injection may occur and is normal. Participants were also reminded that they may receive either 2 local anaesthetics or a placebo and a local anaesthetic (or vice versa). On the second occasion, both messages were repeated. At no stage did any person in contact with the participants mention expected levels of response or criteria for progression to the neurotomy treatment. If participants asked this question, they were reminded that the double-blind nature of the study precluded staff from discussing this topic.

Because of the innervation of the Z joints (from 2 spinal levels), diagnostic injections were performed on the 2 nerves that supply the symptomatic joint, or a maximum of 3 joints (4 nerves) unilaterally and 2 joints (3 nerves) bilaterally in the case of a more widespread presentation. The decision on what joints to inject or “block” was made at initial assessment on history, physical examination, and investigation findings (see inclusion criteria) and reconfirmed on the day of the injections. The target joints from clinical examination were located by fluoroscopy using the approach previously described with injections performed at relevant levels.^4^ For specific details, see Ref. 8. Participants were then allowed to ambulate briefly and then rest seated for 20 minutes, after which they were able to move ad libitum for the remainder of their time at the clinic. Measurement of expected pain relief (% reduction in pain expected after injection) and desire for symptom reduction (0–10 numeric rating scale) were taken immediately before the injections. Primary and secondary measures were taken at ten minutes, 1 hour and then hourly for 7 hours after injection to mirror contemporary practice. Participants were also asked to provide their best assessment of group allocation at 60 minutes after injection.

### 2.4. Outcomes and analysis

The primary outcome measure was pain severity, measured on a (0–10 numeric rating scale [NRS]). Anxiety level was concurrently measured using a 0 to 10 numeric rating scale, as was desire for pain relief (0–10 NRS). Expected benefit was assessed in terms of percentage reduction in pain. This was a pragmatic measure that was designed to mirror the standard injection selection process (whereby in this specific clinical practice, a positive response to an injection is discussed between clinician and participant in terms of percentage of improvement).

Magnitude of placebo responses was evaluated using a repeated-measures analysis of variance. Response rate was reported using frequency analysis. Expected pain reduction, anxiety level, and desire for pain relief were analysed in a categorical manner: high responders being those reporting >50% reduction in pain, and low responders reporting ≤50% for the relevant scale. T-tests were used to compare mean values in the primary pain measure between these groups. This was based on predetermined response categories used after injection therapy and in a pragmatic clinical manner. A regression analysis was also performed to assess relationships between variables. Hierarchical regression analyses were conducted to assess the interaction between expectancy, anxiety, and desire for relief. Alpha values of 0.05 were used for significance. Secondary analyses were aimed at assessment in only those participants who were “responders,” to better characterize this group.

### 2.5. Randomisation and blinding

Computer-generated randomisation was conducted according to a 2:2:1 schedule. The control group had fewer participants randomised to it based on the planned analyses. The randomisation was concealed in sealed, opaque envelopes. The drugs were prepared by an independent clinical nurse not involved in the procedure or the follow-up. At no stage during part 1 were the medical practitioners or nursing staff involved in the procedure aware of a participant's group allocation, minimising any possible bias. A research nurse who was blinded to allocation collected data each day.

## 3. Results

### 3.1. Participant flow

Of the 120 participants randomised in this trial, 9 decided not to commence after randomisation (7 changed their decision on the basis of not wanting the interventional process and 2 withdrew due to limited pain). Of the 111 participants remaining, 4 were excluded before the first block due to having no pain on the day. The remaining 107 (89%) participants commenced the block process. Fourteen participants withdrew between the first block and the end of measurement of pain after the second block (of the 14, 5 received placebo on their first block, and the remainder (9) received local anaesthetic). Ninety-three (78%) participants completed both blocks on the 2 separate days, and this was the final number analysed (Fig. 1). The final number completing both blocks in groups 1 and 2 (one of which would be a placebo) was 82. A further 11 were in the control group (group 3) and received local anaesthetic on both occasions.

### 3.2. Baseline characteristics

Baseline characteristics are presented in Table 1. These are presented in comparison with published normative data for almost 5,000 patients presenting to the same tertiary level chronic pain clinic. This provided an indication of how representative the participants were of a chronic pain clinic population. Of specific note is the mean duration of pain (115 months, or over 9 years) and average intensity of pain (5.6/10 on the NRS).

### 3.3. Outcomes and analyses

#### 3.3.1. Magnitude and response to placebo

Of the 107 participants who commenced the first injection, 96 (groups one and 2) were to receive a placebo injection on 1 of 2 visits. Of these 96 participants, 5 were withdrawn before any block. Ninety-one proceeded to first injection, and 9 participants completed a single block before withdrawal, of which 5 received placebo and were included (total placebo number = 87).

**Figure 2.**
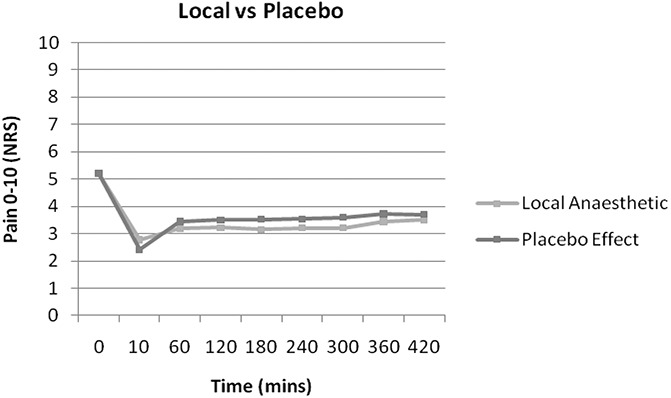
Magnitude of response to local anaesthetic and placebo.

Overall, there was a significant response to placebo from baseline (5.2/10, SD = 2). Repeated-measures analysis confirmed that this was significant at every time interval (*P* = 0.01) (Fig. 2). The initial reduction in pain after placebo was 54% (SD = 43%) (mean reduction of 2.8, 95% CI [2.3–3.3]). The effect tapered to 29% (SD = 64%) pain reduction at 7 hours (a mean reduction of 1.5, 95% CI [0.9–2.1]). In comparison, although not the focus for this part of the study, response to local anaesthesia was slightly less, with a mean 44% reduction in pain (SD = 50), with each period statistically significant (*P* = 0.01). Direct statistical comparison between groups was not possible because the groups were not independent.

**Figure 3.**
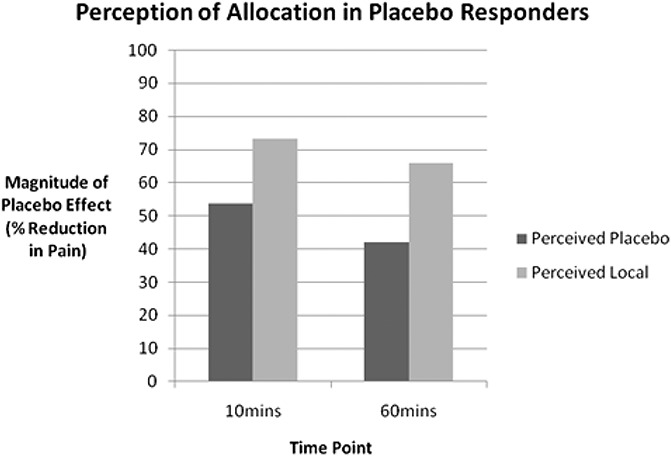
Magnitude of response based on perception of injection content.

Perception of allocation was measured at 10 and 60 minutes after injection to assess whether this perception affected magnitude of response to placebo (Fig. 3). At 10 minutes after injection, response to placebo was larger in those participants who believed they had received local anaesthetic (n = 53) (73%, SD = 28% vs 53%, SD = 28, *P* = 0.014) rather than a placebo (n = 34). A similar statistically significant pattern was seen at 60 minutes (65%, SD = 23% vs 42%, SD = 24, *P* = 0.001). Among those who believed they had received a placebo, the mean response to placebo was a greater than 40% reduction in pain.

The percentage of participants responding to placebo (regardless of magnitude) was initially very large (70 participants, or 78% response rate). At 7 hours, 50 participants (65%) had still reported significantly reduced pain to placebo (when compared with baseline).

A planned secondary analysis of the above data was conducted after placebo nonresponders were removed, thereby removing the effect of participants who reported increased pain, further characterising only those who had positive response to placebo. This demonstrated much larger response to placebo (67% reduction in pain at 10 minutes SD = 29 and 56% reduction at 7 hours SD = 30) with smaller variance and more stability over time. This represented an initial effect size after injection (10 minutes) (Cohen's d) of 1.36 in all participants and 1.85 in only placebo responders (78% of the study population), and large and sustained effect size at 7 hours (1.64) in placebo responders compared with 0.73 when nonresponders were included.

#### 3.3.2. Role of expectancy, desire, and anxiety

Participants were divided into 2 groups based on preinjection expected pain reduction (as a percentage). There was no statistically significant difference between those who expected low (1%–50%) or high (51%–100%) reduction in pain at 10 minutes (63% reduction in pain SD = 30.13 in the low expectancy group and 72% reduction in the high group, SD = 30.11, *P* = 0.229). This effect was repeated at 60 minutes (31% in low expectancy, SD = 44% vs 46% in high group, *P* = 0.314). However, a significant difference was found at 7 hours (10% reduction in low expectancy, SD = 56% vs 57% in high expectancy, SD = 32, *P* = <0.001). These findings were supported by correlations (Pearsons) between high vs low groups that were not significant at 10 minutes (*r* = 0.3, *P* = 0.28) or 60 minutes (*r* = 0.16, *P* = 0.169) but were significant at 7 hours (*r* = 0.43, *P* = 0.001). Taken together, expectancy of benefit (low or high) was not associated with significant difference in initial pain reduction after placebo.

On the basis of these results, a post hoc analysis was conducted to see whether this baseline measure of expected pain reduction was associated with stability of response to placebo. This was based on the findings of a more stable response to placebo when initial nonresponders were removed, and that baseline expectancy was only significant to placebo analgesia at the 7-hour stage. Accordingly, a change score in pain (%) between the levels at 10 minutes after injection and 7 hours was calculated, using the same statistical analysis method. Mean change in pain score between 10 minutes after injection and 7 hours was −107% (participants had worsening or return of preinjection pain) in those with low baseline expectancy. By contrast, those with high expectancy had a change score of +8%, demonstrating a maintenance of effect. Correlation analysis confirmed this relationship (*r* = 0.405, *P* = 0.01).

The same analytic method was performed with anxiety scores. Participants were grouped based on their change in anxiety (from preprocedure level to postprocedure level). We were therefore able to assess response to placebo in those who we categorised as having either low change (1%–50% reduction) or high change (51%–100% reduction) in anxiety. This was to assess the relationship between change in anxiety and response to placebo. There was no statistical difference in response to placebo in those participants who reported low or high change in anxiety, respectively, (10 minutes: 64% [32] response to placebo in low anxiety vs 69% [29] *P* = 0.68, 60 minutes: 29% [22] vs 37% [71] *P* = 0.553, and 7 hours: 28% [32] vs 38% [42]). Although there were no statistically significant differences found, there was substantial variance and a trend for larger responses to placebo in those with larger changes (reductions) in anxiety. Correlation was not significant at 10 and 60 minutes; however, there was a correlation between anxiety reduction and magnitude of response to placebo at 7 hours (*r* = 0.29, *P* = 0.03).

The planned analysis of desire for pain relief was unable to be performed. At the time of this trial, the construct had not been applied to clinical pain, and there was no measure of this construct available for this setting. The mean desire for pain relief (0–10) was 9.18/10 (2.03). Correction for outliers (by conversion to *Z* score) only removed 4 participants, resulting in a mean of 9.56 (1.03). Most participants, therefore, reported scores close to the maximum value, markedly skewing the measure. This affected both the examination of desire as a single construct and hierarchical regression due to lack of correlation.

The predetermined variable interaction analysis was therefore only conducted on expectancy and anxiety, and only at 7 hours after injection (as this was the only time point where statistically significant correlations occurred in both). Baseline expectancy, change in anxiety, and the interaction variable (expectancy × anxiety) were entered into the hierarchical regression. Response to placebo (difference from baseline to 7 hours) served as the predictor variable (Table 2). Expectancy was entered first, accounting for 16% of the variance in response to placebo (F(8.778), *P* = 0.001). When anxiety was added, this accounted for a further 10% of the variance (F(7.835), *P* = 0.001). The addition of the interaction variable (expectancy × anxiety) accounted for a further 7% (F(7.34), *P* = 0.01). Together, the entire model accounted for 32% of the variance in response to placebo.

**Table 2:**
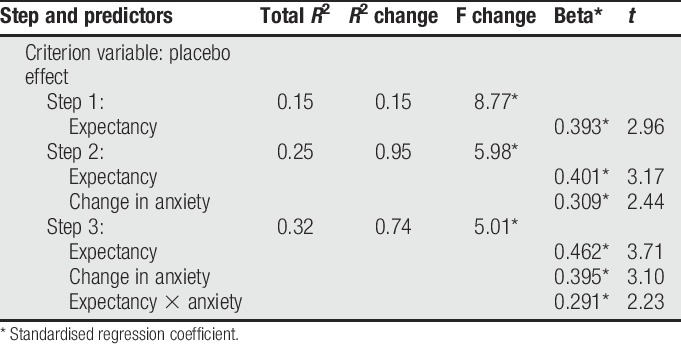
Interaction analysis between expectancy and anxiety.

## 4. Discussion

This clinical trial demonstrated significant responses (both in magnitude and rate) to placebo injections with participants reporting CLBP. These effects mirrored the effect of local anaesthesia, although comparison was not an intended aim of the study. There was a significant amount of variance in the data, and when placebo nonresponders were removed (to characterise the magnitude of response in placebo responders), the effect size was very large, in fact, larger than effects quoted in meta-analyses^1,36^ and key papers in clinical pain.^17^ Response rates were also high when compared with published literature; at 78%, this was much larger than the seminal proto-meta-analysis by Beecher (30% response rate),^1^ and other key papers in clinical pain (39% in Ref. 16) and experimental pain (56% in Ref. 22). Interestingly, the duration of response mirrored the informed consent process and clinical context, whereby participants were told that the injections were a short-term diagnostic measure, and that pain would only be measured for 7 hours. This finding is consistent with the context specific nature of responses to placebo.^8^

An interesting additional finding was the perception of allocation. Our hypothesis was that response to placebo would be larger when participants perceived that they had been given local anaesthesia. This hypothesis was confirmed, however, of note was the large response magnitude to placebo in participants who perceived correctly that they had received placebo. Although it is difficult to know which comes first, the experience and appraisal of the treatment effect and then the perception of allocation or vice versa, the finding that response to placebo can be large despite participants acknowledging their receipt of a placebo is very significant. Although no qualitative data were obtained, this finding is partly supported by recent open-label placebo trials and remains an interesting area for future research.^15^

The roles of expectancy, anxiety, and desire for pain relief were also assessed. The aim was to replicate and extend the findings of several trials that used an experimental pain model in a clinical population.^37–39^ Expectancy of pain relief was associated with larger responses to placebo, but this was only significant at 7 hours. A post hoc analysis demonstrated that level of baseline expectancy was associated with stability of response to placebo after the effect was generated. That is, most participants had an initial response, but this response was only stable (ie, it did not decay) in those with high baseline expectancy of pain reduction. Unfortunately, we only assessed baseline expectancy rather than expectancy as a dynamic construct (ie, as time elapsed in the follow-up period). Our results indicate that expectancy may be associated with magnitude of placebo responses to injections in CLBP. Expectancy may also be a dynamic determinant of the stability of response to placebo once the effects are generated.

Our attempt to further characterise the role of anxiety and desire, through assessment of an expectation-desire-emotion model was limited in 2 key ways.^26^ First, there was a significant degree of variance in measures of anxiety. Despite relatively large numbers, and a trend that placebo responses were larger in those with greater reductions in anxiety, this was not statistically significant. This is an important characterisation of this variable in the specific context of day procedure injections. Second, as previously mentioned, the scores on the 0 to 10 desire for pain reduction measure were extremely skewed. This meant that we were unable to assess desire alone or in the model. Although on one hand, this was a limitation of the study; on the other hand, this is an important extrapolation of this construct to response to placebo in a CLBP setting (noting, we benchmarked our participants to a large published data set characterising participants who seek tertiary level chronic pain management care). In this setting, desire for relief is very high, and it is difficult to assess its relative contribution to the magnitude of placebo responses in this setting. Our regression model was able to characterise the important roles of expectancy, change in anxiety, and the interaction between the two at 7 hours after injection. It is unclear why the significance only occurred at the delayed time point. One possible explanation is that these constructs are particularly important in the maintenance (rather than generation of) placebo effects. This explanation is supported by our results and in part by a small study which demonstrated that a measure of midpoint expectancy was better associated with placebo response than an initial baseline measure.^38^ This is a potentially critical piece of information when considering how placebo mechanisms operate, and that 2 discrete phases may exist, first a generation phase, followed by a maintenance phase. This will be an important area of future research because we are only able to provide some initial support for this hypothesis. Nevertheless, the roles of expectancy and (change in) anxiety have been supported and extended in this particular clinical population.

It is increasingly recognised that there is not one “placebo effect,” rather there are many, and these effects are context specific.^9,27^ It is therefore important to characterise placebo effects in specific contexts, particularly in various clinical settings where research on placebo effects is lacking. The major strengths of this work are that it has been able to characterise placebo effects in a specific (but common) chronic pain patient population. Specifically, we have characterised these effects after injections, in a setting where response to placebo can be deemed to be a response that is not genuine and can shape ongoing treatment decisions.^5^ However, there are limitations in the study that must be acknowledged. These include not using a group that received 2 placebos. This would have controlled for the possible influence of guessing by the participants. But because of ethical constraints, such a condition could not be supported in a clinical population. It might also have strengthened the design if pain ratings had been monitored over an extended baseline phase (up to 7 hours in this case) to test for the natural history of the pain ratings in this population, and this should be considered in future trials, consistent with n = 1 study designs.^20^ The limited time evaluated (up to 7 hours) might be seen as too restrictive, but as noted earlier, this is the standard clinical protocol used at the treatment centre.

Furthermore, we have been able to support previous findings for the role of expectancy and anxiety, and the interaction between the two, with some evidence presented as to the potential significance of these as dynamic constructs rather than baseline predictors. On one hand, the desire measure was limited due to a skewed data set, but on the other hand, this has characterised the measure in a particular population. That is, this population had a strong desire for pain relief. We were able to also show the interesting finding that participants reported large reductions in pain while believing it was a placebo that had been given. Taken together, this work is generalizable to broader chronic pain conditions; however, given the specific nature of the clinical context, our work needs to be assessed in different conditions, particularly over a longer duration.

## 5. Conclusion

Placebo injections in the setting of diagnosis for CLBP yield both large rates and magnitude of analgesic responses compared with published literature. Expectation of pain reduction and change in anxiety were important psychological determinants, with support for the role of expectancy as a dynamic modulator of placebo effects rather than a baseline predictor. In this study, participants reported large responses to placebo despite the belief that they had received a placebo injection. Future research will allow for this work to be extended to different clinical pain populations and over a longer duration.

## Disclosures

The authors have no conflict of interest to declare.

This research was supported by an Australian and New Zealand College of Anaesthetists (ANZCA) Project Grant.
